# Ethnic difference in the prevalence of angina pectoris in Sami and non-Sami populations: the SAMINOR study

**DOI:** 10.3402/ijch.v73.21310

**Published:** 2014-01-10

**Authors:** Bent-Martin Eliassen, Sidsel Graff-Iversen, Marita Melhus, Maja-Lisa Løchen, Ann Ragnhild Broderstad

**Affiliations:** 1 Faculty of Health Sciences, Department of Community Medicine, Centre for Sami Health Research, UiT The Arctic University of Norway, Tromsø, Norway; 2 Norwegian Institute of Public Health, Nydalen Oslo, Norway; 3 Faculty of Health Sciences, Department of Community Medicine, UiT The Arctic University of Norway, Tromsø, Norway; 4 Department of Medicine, University Hospital of Northern Norway, Harstad, Norway

**Keywords:** cardiovascular disease, Arctic, Sami, indigenous peoples, Norway, minority

## Abstract

**Objective:**

To assess the population burden of angina pectoris symptoms (APS), self-reported angina and a combination of these, and explore potential ethnic disparity in their patterns. If differences in APS were found between Sami and non-Sami populations, we aimed at evaluating the role of established cardiovascular risk factors as mediating factors.

**Design:**

Cross-sectional population-based study.

**Methods:**

A health survey was conducted in 2003–2004 in areas with Sami and non-Sami populations (SAMINOR). The response rate was 60.9%. The total number for the subsequent analysis was 15,206 men and women aged 36–79 years (born 1925–1968). Information concerning lifestyle was collected by 2 self-administrated questionnaires, and clinical examinations provided data on waist circumference, blood pressure and lipid levels.

**Results:**

This study revealed an excess of APS, self-reported angina and a combination of these in Sami relative to non-Sami women and men. After controlling for age, the odds ratio (OR) for APS was 1.42 (p<0.001) in Sami women and 1.62 (p<0.001) for men. When including relevant biomarkers and conventional risk factors, little change was observed. When also controlling for moderate alcohol consumption and leisure-time physical activity, the OR in women was reduced to 1.24 (p=0.06). Little change was observed in men.

**Conclusion:**

This study revealed an excess of APS, self-reported angina and a combination of these in Sami women and men relative to non-Sami women and men. Established risk factors explained little or none of the ethnic variation in APS. In women, however, less moderate alcohol consumption and leisure-time physical activity in Sami may explain the entire ethnic difference.

The Sami are an indigenous people whose traditional settlement area (Sápmi) stretches across the northern parts of Norway, Sweden and Finland, and Russia's Kola Peninsula. The Sami languages belong to the Finno-Ugric language group. There is limited knowledge concerning the burden of cardiovascular diseases in the Sami population of Norway and whether there are differences in the morbidity of chronic non-communicable diseases among the Sami relative to the non-Sami population.

Since the Second World War, the Sami and non-Sami have undergone lifestyle changes, including an increase in sedentary lifestyles and changes in diet (1). A high prevalence of obesity among Sami women compared with Norwegian women has been found (1). A recent study observed a somewhat higher apoB/apoA-1 ratio and cholesterol level in middle-aged Sami men and women compared with non-Sami men and women (2). These 2 studies were both SAMINOR studies. In the first Finnmark study (3) conducted in 1974–75, Sami/Finnish men aged 35–49 years had on average a 40% higher cardiovascular risk score compared with Norwegians. The score was based on sex, serum total cholesterol, systolic blood pressure and current cigarette smoking (3). Despite this risk profile, the prevalence of self-reported myocardial infarction was considerably lower in the Sami compared with the Norwegian population (3). Since then, prevalence and follow-up data have shown no or only minor differences in risk factors and risk of cardiovascular disease (4–8). In terms of mortality from cardiovascular disease, conflicting results have been presented (9, 10). Various definitions of Sami ethnicity have been used in these studies.

Detection and treatment of angina can help prevent or delay an acute myocardial infarction. Assessing the population burden of angina pectoris and exploring potential ethnic disparity in its distribution with regard to traditional risk factors in areas with both Sami and non-Sami populations is thus relevant. The primary objective of this study was to assess the relationship between ethnicity and the prevalence of angina pectoris symptoms (APS) in the rural population of northern Norway by using a 2-item version of the Rose angina questionnaire (RAQ). If differences were found between Sami and non-Sami populations, we aimed at evaluating the role of established cardiovascular risk factors as mediating factors for such differences. We also measured the burden of self-reported angina pectoris alone and in combination with the RAQ.

## Methods

As part of the Norwegian Institute of Public Health's (NIPH) health survey in Finnmark, the Centre for Sami Health Research conducted in 2003–2004 a population-based survey in areas with Sami and non-Sami populations (SAMINOR) (11), also including areas outside Finnmark. The age cohorts included in SAMINOR were 30 and 36–79 year-olds. All eligible residents in the selected municipalities registered in the Central Population Register were invited regardless of ethnic background (N=27,987). Of these, 16,865 (60.3%) participated. The response rate in the age group 36–79 years (n=16,538) was 60.9%. The population was almost exclusively rural. The Regional Committee for Medical Research Ethics approved the study, and the participants gave written informed consent.

Only participants who gave consent to medical examination and who responded to the initial and main questionnaires were included in the analysis (11). Those aged 30 years (low response rate) and recent immigrants were excluded from the analyses; recent immigrants were responders who were born abroad and answered “other” to the first 2 questions on ethnicity (see questionnaires). The total number for the subsequent analysis was 15,206 men and women aged 36–79 years (born 1925–1968).

### Questionnaires

Two self-administrated questionnaires were used to collect information on ethnicity and lifestyle. Ethnicity was measured by the following: *What language(s) do/did you, your parents and your grandparents use at home?* The response categories were “Norwegian,” “Sami,” “Kven” or “other.” Kvens are descendants of Finnish settlers who immigrated to northern Norway in the 1700s and 1800s (12). Providing the same response options we also asked: *What is your, your father's and your mother's ethnic background?* The respondents also reported whether they considered themselves to be Norwegian, Sami, Kven or other (self-perceived ethnicity). For all these questions multiple answers were allowed. Based on these variables, we generated 2 ethnic categories, that is, Sami and non-Sami. The Sami category included respondents reporting at least one Sami identity mark (Sami language spoken by the respondent or at least one parent or grandparent, or Sami ethnic background or self-perceived Sami ethnicity) while Norwegians and Kvens (n=1,137) were included in the non-Sami group. These variables are described in detail by Lund et al. (11).

APS were measured by the following questions: 1) *Do you get pain or discomfort in the chest when walking up hills or stairs, or walking fast on level ground?* (yes/no) and 2) Do you get such pain or discomfort even if you are resting? (yes/no). Missing values (n=526 and 2,240, respectively) were considered negative responses.

We defined APS as a positive response to the former and a negative one to the latter (13). Positive responses to both questions were considered as not having APS.

Self-reported angina was measured by the question: *Do you have, or have you had angina pectoris (heart cramp)?* Missing values (n=636) were considered negative responses.

Waist circumference (WC) was measured at the umbilicus to the nearest centimetre with the individual standing and breathing normally. The methods used and procedures followed for the measurement of blood pressure, total cholesterol, HDL cholesterol, triglycerides and glucose are described in detail elsewhere (2). LDL cholesterol was computed by using the Friedwald formula [LDL=total cholesterol – HDL cholesterol – (Triglycerides*0.45)]. All blood samples in the SAMINOR study were non-fasting. We used self-reported diabetes and information about anti-diabetic medication to define diabetes. In addition, non-fasting blood glucose level ≥11.1 was defined as having diabetes (14).

The following question measured family history of myocardial infarction: *Tick off relatives who have, or have ever had, any of the following conditions, and report the age of when they got the illness*: “Myocardial infarction before the age of 60 years.” This is a conventional cut-off (15). The possible response categories were: “Mother,” “Father,” “Sister,” “Brother,” “Children” and “No one.” Observations providing positive answers but reporting the age of 60 or more for when their relative(s) got the illness (n=204) were given a negative response. Those giving a negative response but reporting age <60 were given a positive response (n=2).

Data on smoking were collected by asking: *Are you currently, or were you previously, a daily smoker*? “Yes, currently,” “Yes, previously,” “Never.” Never smokers (n=37), current smokers (n=86) and observations with missing data on smoking status (n=14) reporting number of years since they stopped smoking were coded as previous smokers.

Education was measured by asking: *How many years of schooling/education have you completed?*

Use of cholesterol-lowering drugs during the past 4 weeks was registered by the following item: *State the name of the medicine and your reason for taking/having taken them (disease, symptoms): Tick one box for each line*. The ATC reference codes for statins were used.

Frequency of alcohol consumption was ascertained by asking: *How often during the last year have you consumed alcohol?* Eight response options ranging from “never consumed alcohol” to “4–7 times per week” were given. We also asked: *To those who have consumed alcohol during the past year: When you drink alcohol, how many glasses or drinks do you normally drink?* From this we produced the following categories: “None,” “A few times,” “Drinking 1–2 units once a week or more often” and “Drinking 3+ units once a week or more often.” Given that the number of units consumed during the past year was greater than 0, “never drinkers” (n=52) and observations with missing info on drinking frequency (n=56) were coded as drinking “a few times.” We were unable to compute “numbers of drinks per day” or “grams of alcohol per day” which are usually the preferred measure (17).

The following question was asked to ascertain physical activity during leisure time: *Exercise and physical exertion in leisure time. If your activity varies much, for example between summer and winter, then give an average. The questions refer only to the past twelve months. (Tick the most appropriate box)*. The possible responses given were: a) “Reading, watching TV or other sedentary activity,” b) “Walking, cycling or other forms of exercise at least 4 hours a week (including walking or cycling to place of work, Sunday-walking, etc.),” c) “Participation in recreational sports, heavy gardening, and so on (note: duration of activity at least 4 hours a week)” or d) “Participation in hard training or sports competitions, regularly several times a week.” Categories c and d were merged as only 204 participants ticked the latter box. This question corresponds well with objective measured physical activity (18).

### Statistical analysis

Means and rates in Tables I and II were tested by 2 sample *t*-tests and Pearson's χ^2^ tests. The prevalence rates in Table III were based on logistic regression estimates. Differences between the rates were tested by performing likelihood ratio tests based on the same regression models. The total rates in Table III were compared with rates produced by direct standardization using the European standard population (19) as reference. Simple tabulation and Pearson's χ^2^ tests were performed to compare self-reported angina and APS (Tables IV and V).Logistic regression (Table VI) was then used to evaluate age, statin use and established CVD risk factors as potential confounding or mediating factors in the association between ethnicity and APS. Such potential effects were assessed by performing forward stepwise regression and evaluating the changes in the effect of ethnicity on APS. Education was excluded from the analyses as no confounding effect was observed.

**Table I:** Characteristics of the female study group by ethnicity. Values are means^a^ or percentages^b^, p-values for differences between ethnic groups (The SAMINOR study 2003–2004)

	Non-Sami	Sami	
		
Variables	n=5,273	n=2,611	p
Age (years)	54.5 (54.2–54.8)	54.2 (53.7–54.6)	0.21
Waist circumference (cm)	85.5 (85.2–85.8)	86.0 (85.6–86.5)	0.06
Triglycerides (mmol/l)^c^	1.35 (1.33–1.37)	1.38 (1.35–1.41)	<0.05
Systolic blood pressure	130.4 (129.8–131.0)	129.7 (128.8–130.5)	0.13
Diastolic blood pressure	73.0 (72.7–73.2)	72.3 (71.9–72.7)	0.01
LDL cholesterol (mmol/l)^d^	3.81 (3.78–3.84)	3.82 (3.78–3.86)	0.70
Total cholesterol (mmol/l)	5.99 (5.96–6.02)	5.98 (5.93–6.02)	0.62
Diabetes (yes)	4.3 (3.7–4.8)	5.0 (4.2–5.9)	0.12
p-Glucose (mmol/l)^c^	5.44 (5.41–5.47)	5.50 (5.46–5.55)	0.02
Statin use (yes)	11.3 (10.4–12.1)	11.9 (10.6–13.1)	0.43
Family history of MI (yes)	24.2 (23.0–25.3)	27.2 (25.5–28.9)	<0.01
Education (≥13 years) (Yes)	34.2 (32.9–35.5)	33.5 (31.6–35.4)	0.56
Smoking			0.38
Never	37.6 (36.3–38.9)	36.4 (34.5–38.2)	
Previous	31.3 (30.1–32.6)	31.0 (29.3–32.8)	
Current	31.1 (29.9–32.4)	32.6 (30.8–34.4)	
Alcohol consumption			<0.001
None	18.1 (17.0–19.1)	28.9 (27.1–30.6)	
A few times	60.2 (58.9–61.6)	57.0 (55.0–58.9)	
1–2 drinks/week	16.7 (15.7–17.7)	9.8 (8.6–11.0)	
≥3 drinks/week	5.1 (4.5–5.7)	4.4 (3.6–5.2)	
Leisure-time physical activity			<0.001
Sedentary	22.3 (21.1–23.5)	26.8 (25.0–28.6)	
Exercise at least 4 hours/week	66.4 (65.1–67.8)	62.2 (60.3–64.2)	
Hard gardening/sports/hard training 4+ hours/week	11.3 (10.4–12.2)	10.9 (9.7–12.2)	

a Tested by 2-sample *t*-test with equal variances.

b Tested by Pearson's χ^2^ test.

c Geometric mean.

d Computed using the Friedwald formula.

**Table II:** Characteristics of the male study group by ethnicity. Values are means^a^ or percentages^b^, p-values for difference between ethnic groups (The SAMIONOR study 2003–2004)

	Non-Sami	Sami	
	
Variables	n=4,746	n=2,576	p
Age (years)	54.8 (54.5–55.1)	55.0 (54.6–55.4)	0.50
Waist circumference (cm)	95.0 (94.7–95.3)	93.2 (92.8–93.6)	<0.001
Triglycerides (mmol/l)^c^	1.61 (1.58–1.63)	1.60 (1.56–1.63)	0.65
Systolic blood pressure	134.2 (133.7–134.7)	134.9 (134.2–135.7)	0.13
Diastolic blood pressure	78.2 (78.0–78.5)	77.9 (77.5–78.3)	0.21
LDL cholesterol (mmol/l)^d^	3.80 (3.77–3.83)	3.87 (3.83–3.92)	<0.01
Total cholesterol (mmol/l)	5.89 (5.86–5.92)	5.98 (5.93–6.02)	<0.01
Diabetes (yes)	4.5 (3.9–5.1)	5.3 (4.4–6.2)	0.14
p-Glucose (mmol/l)^c^	5.61 (5.57–5.64)	5.63 (5.58–5.68)	0.50
Statin use (yes)	14.6 (13.6–15.6)	14.9 (13.5–16.3)	0.71
Family history of MI (yes)	23.0 (21.8–24.2)	26.1 (24.4–27.8)	<0.01
Education (≥13 years) (yes)	31.4 (30.0–32.7)	26.6 (24.9–28.4)	<0.001
Smoking			<0.05
Never	28.4 (27.1–29.7)	25.5 (23.9–27.2)	
Previous	41.5 (40.1–42.9)	42.5 (40.6–44.4)	
Current	30.2 (28.9–31.5)	32.0 (30.2–33.8)	
Alcohol consumption			<0.001
None	9.1 (8.3–9.9)	13.3 (11.9–14.6)	
A few times	58.1 (56.7–59.5)	60.6 (58.7–62.5)	
1–2 drinks/week	18.4 (17.3–19.5)	13.4 (12.0–14.7)	
≥3 drinks/week	14.4 (13.4–15.4)	12.8 (11.5–14.1)	
Leisure-time physical activity			0.35
Sedentary	23.0 (21.8–24.3)	24.5 (22.8–26.3)	
Exercise at least 4 hours/week	55.4 (54.0–56.9)	54.8 (52.7–56.8)	
Hard gardening/sports/hard training 4+hours/week	21.5 (20.3–22.8)	20.7 (19.1–22.3)	

a Tested by 2-sample *t*-test with equal variances.

b Tested by Pearson's χ^2^ test.

c Geometric mean.

d Computed using the Friedwald formula.

**Table III:** Age-specific and overall prevalence (%)^a^ of angina pectoris symptoms (APS), self-reported angina, and APS and self-reported angina combined by sex and ethnic groups (n=15,206) (The SAMINOR study 2003–2004)

	Non-Sami			Sami			
			
	n	%	95% CI	n	%	95% CI	p^b^
APS							
*Women*							
36–49	1,947	2.8	2.2–3.7	1,009	3.4	2.4–4.7	0.42
50–59	1,557	4.5	3.6–5.7	795	6.3	4.8–8.2	0.07
60–69	1,110	7.2	5.8–8.9	496	10.1	7.7–13.1	0.06
70–79	659	11.7	9.5–14.4	311	17.7	13.8–22.3	0.01
Total^c^	5,273	4.6	4.0–5.2	2,611	6.3	5.5–7.3	<0.001
*Men*							
36–49	1,657	1.9	1.3–2.7	875	2.7	1.9–4.1	0.16
50–59	1,487	3.8	3.0–4.9	850	9.2	7.4–11.3	<0.001
60–69	1,041	8.6	7.0–10.4	536	12.5	10.0–15.6	0.01
70–79	561	17.1	14.2–20.5	315	18.7	14.8–23.4	0.55
Total^c^	4,746	4.4	3.9–5.1	2,576	6.9	6.0–8.0	<0.001
Self-reported							
*Women*							
36–49	1,947	0.4	0.2–0.8	1,009	0.3	0.1–0.9	0.62
50–59	1,557	2.1	1.5–2.9	795	4.7	3.4–6.4	<0.001
60–69	1,110	7.5	6.1–9.2	496	10.5	8.1–13.5	<0.05
70–79	659	16.1	13.5–19.1	311	27.3	22.7–32.6	<0.001
Total^c^	5,273	1.9	1.6–2.3	2,611	3.3	2.7–4.1	<0.001
*Men*							
36–49	1,657	1.0	0.6–1.6	875	1.0	0.5–2.0	0.99
50–59	1,487	5.2	4.2–6.4	850	8.4	6.7–10.4	<0.01
60–69	1,041	12.8	10.9–15.0	536	17.0	14.0–20.4	<0.05
70–79	561	22.1	18.9–25.7	315	24.1	19.7–29.2	0.50
Total^c^	4,746	4.9	4.3–5.5	2,576	6.5	5.6–7.5	<0.001
Combined							
*Women*							
36–49	1,947	3.1	2.4–4.0	1,009	3.7	2.7–5.0	0.40
50–59	1,557	6.2	5.1–7.5	795	9.2	7.4–11.4	<0.01
60–69	1,110	12.3	10.5–14.4	496	17.3	14.3–20.9	<0.01
70–79	659	23.1	20.0–26.4	311	33.8	28.7–39.2	<0.001
Total^c^	5,273	6.2	5.5–6.9	2,611	9.0	7.9–10.2	<0.001
*Men*							
36–49	1,657	2.7	2.0–3.6	875	3.7	2.6–5.1	0.20
50–59	1,487	8.1	6.9–9.6	850	14.7	12.5–17.3	<0.001
60–69	1,041	17.9	15.7–20.3	536	23.9	20.5–27.7	<0.01
70–79	561	31.7	28.0–35.7	315	33.0	28.0–38.4	0.70
Total^c^	4,746	8.3	7.5–9.2	2,576	11.7	10.5–13.0	<0.001

a Prevalence rates from logistic regression estimates.

b p-value from likelihood ratio test for difference between non-Sami and Sami.

c Age adjusted.

**Table IV:** Angina pectoris symptoms (APS) relative to self-reported angina in women (The SAMINOR study, 2003–2004)

		Self-reported angina		
			
Non-Sami (p<0.001)^a^		No (%)	Yes (%)	Total
Symptoms	No	4,828 (95.7)	163 (71.2)	4,991
	Yes	216 (4.3)	66 (28.8)	282
	Total	5,044 (100)	228 (100)	5,273
Sami (p<0.001)^a^		Self-reported angina		
		No (%)	Yes (%)	Total
Symptoms	No	2,310 (94.9)	112 (63.3)	2,422
	Yes	124 (5.1)	65 (36.7)	189
	Total	2,434 (100)	177 (100)	2,611

a Pearson's χ^2^ test.

**Table V:** Angina pectoris symptoms (APS) relative to self-reported angina in men (The SAMINOR study 2003–2004)

		Self-reported angina		
			
Non-Sami (p<0.001)^a^		No (%)	Yes (%)	Total
Symptoms	No	4,216 (95.9)	257 (73.2)	4,473
	Yes	179 (4.1)	94 (26.8)	273
	Total	4,395 (100)	351 (100)	4,746
Sami (p<0.001)^a^		Self-reported angina		
		No (%)	Yes (%)	Total
Symptoms	No	2,187 (93.9)	161 (65.2)	2,348
	Yes	142 (6.1)	86 (34.8)	228
	Total	2,329 (100)	247 (100)	2,576

a Pearson's χ^2^ test.

**Table VI:** Odds ratios (OR) and 95% confidence intervals (CI) for the difference in angina pectoris symptoms (APS) between non-Sami and Sami by sex (The SAMINOR study 2003–2004)

	Women			Men		
		
	OR	p	95% CI	OR	p	95% CI
Model 1^a^	n=7,884			n=7,322		
Non-Sami	Ref			Ref		
Sami	1.42	<0.001	1.17–1.72	1.62	<0.001	1.34–1.95
Model 2^b^	n=7,763			n=7,244		
Non-Sami	Ref			Ref		
Sami	1.38	0.001	1.13–1.68	1.69	<0.001	1.39–2.05
Model 3^c^	n=6,859			n=6,648		
Non-Sami	Ref			Ref		
Sami	1.24	0.06	1.00–1.54	1.78	<0.001	1.45–2.19

Controlling for:

a age.

b age+previous and current smoking, waist circumference, systolic blood pressure, triglycerides, LDL cholesterol, diabetes, family history of myocardial infarction, total cholesterol and use of statins.

c as b+moderate alcohol consumption and leisure-time physical activity.

A doctor's diagnosis is usually followed by lipid-lowering treatment and life-style changes. To avoid some of the challenges of invalid temporal relationships (20) between the outcome and the independent variables, we chose APS as the outcome as many reporting APS were assumed possible undiagnosed cases of angina.

Pulmonary diseases may produce chest symptoms upon exertion. Potential confounding attributable to self-reported chronic bronchitis/emphysema/obstructive pulmonary disease, asthma, psychological troubles sought help for and use of anti-hypertensive drugs was assessed by including these items in the regression model. Binge drinking, and intensity and duration of smoking [pack-years in current smokers and time since smoking cessation in previous smokers (16)] were also included in the regression to assess any residual confounding effects. We also performed the regression with and without the ad-hoc imputation to assess if the imputations of negative angina cases influenced estimates and results in Table VI.

An alternative categorization of Sami ethnicity was assessed by dichotomizing Sami ethnicity, that is, Sami I (Sami language used as home language by all grandparents, parents and the participant) and Sami II (at least one Sami identity mark, i.e. Sami language spoken by the respondent or at least one parent or grandparent, or Sami ethnic background or self-perceived Sami ethnicity). In addition, alternative analyses were made with regard to geography by performing the analyses separately for inland and coastal situated municipalities.

All statistical analyses were performed using STATA version 12.1 (StataCorp, College Station, TX).

## Results

Tables I and II display the levels of selected risk factors by ethnicity in women and men, respectively. In women, statistically significant (p<0.05) but small differences between non-Sami and Sami were found for triglycerides, diastolic blood pressure, p-glucose, family history of myocardial infarction, moderate alcohol consumption and leisure-time physical activity. The most striking differences were found in moderate alcohol consumption and leisure-time physical activity wherein lower levels were observed among the Sami.

In men, statistically significant (p<0.05) differences were found for WC, total cholesterol, LDL cholesterol, family history of myocardial infarction, years of education, smoking and moderate alcohol consumption. However, these differences were also small.

Table III displays age-specific and total age-adjusted prevalence rates of APS, self-reported angina and a combination of these by sex and ethnic groups. Overall ethnic differences were found in women and men for all 3 measures (p<0.001), and a pattern of higher estimates in the Sami population was revealed in nearly all age strata in both women and men. The combined burden of angina pectoris was 6.2% and 9.0% in non-Sami and Sami women, respectively. In men, the rate for non-Sami was 8.3% and for Sami 11.7%. The prevalence rates produced by logistic regression estimates overlapped considerably with rates produced using direct standardization (data not shown).

Crude numbers of persons with APS relative to self-reported angina pectoris are presented by sex and ethnicity in Tables IV and V. In non-Sami women with self-reported angina, 66 of 228 (28.8%) had APS, compared to 65 of 177 (36.7%) among Sami women. Slightly lower percentages were seen in men. In women with a negative or no report of angina pectoris diagnosis, the percentage with APS was 4.3% in non-Sami and 5.1% in Sami. Among men without self-reported angina pectoris, the percentage with APS was 4.1% in the non-Sami and 6.1% in the Sami.

Odds ratios (OR) for APS are presented in Table VI. The OR for Sami women was 1.42 (p<0.001) and for men 1.62 (p<0.001) after controlling for age (Model 1). When including relevant biomarkers and conventional risk factors, little change was observed (Model 2). When also controlling for moderate alcohol consumption and leisure-time physical activity (Model 3), the OR in women was reduced to 1.24 (p=0.06). Little or none change was observed in men. Overall, results were not affected by confounding attributable to pulmonary disease and psychological troubles, and no residual confounding due to intensity and duration of smoking, and binge drinking was observed. Furthermore, the ad-hoc imputation of negative cases of APS and the alternative categorization of ethnicity did not affect the end result (data not shown).

## Discussion

This study showed an excess burden of self-reported angina and APS in Sami women and men compared with a non-Sami population in the same region. In women, the excess risk was explained by lower moderate alcohol consumption and a more sedentary lifestyle in Sami compared with non-Sami. If the objective is to assess lifetime angina pectoris, our findings suggest that a combination of self-reported angina and angina measured through the 2-item RAQ is important. A relatively high overall response rate (61%) and a large sample have enabled an in-depth comparative analysis of APS in Sami and non-Sami populations. With the exception of Nordland county, the sample is representative for the general rural population in the region (2).

The overall validity of the RAQ has varied depending on the gold standard applied and poor sensitivity and specificity among women, especially younger women, have been reported (21). Chronic bronchitis, bronchial asthma, pulmonary disease and symptoms, depression, anxiety and non-cardiac pain may be reasons for positive RAQ outcomes (22–25). Nevertheless, Graff-Iversen et al. (24) found that a 3-item version of the RAQ predicted mortality from ischaemic heart disease (IHD) in Norwegian women and men aged 40–49 years. Furthermore, studies among men have consistently found a strong association of positive outcome angina as measured by the RAQ and IHD mortality (24).

The validity of our 2-item version of the RAQ, including the term “chest discomfort” has to our knowledge not been assessed with regard to relevant gold standards. A positive outcome by this version of the RAQ may also reflect other diseases than the ones mentioned, e.g. gallbladder disease, cancer and gastric ulcer (26–28). It is nevertheless plausible to assume that the symptoms in the ages above 45–50 years reflect angina in the majority of cases. Regardless of its validity, our 2-item RAQ is of public health interest beyond that of being an IHD screening tool as it also reflects the population burden of pain, discomfort and functional limitations.

Lampe et al. (29) have reported substantial agreement between self-reported doctor diagnosed angina and medical case history in British men. However, a recent review has indicated that self-reported stable angina pectoris may be less reliable than other self-reported cardiovascular diagnoses (21). Whether the excess burden of angina and its symptoms in this study is due to underreporting in non-Sami or over-reporting in Sami is unknown. Language problems are unlikely as only 1.6% of the participants chose to use the Sami version of the questionnaire.

The finding that a minority of those reporting angina also reported APS (Tables IV and V) may be due to treatment. It may however also suggest confounding attributable to other diseases; we found no effect on our point estimates (Table VI) when adjusting for self-reported pulmonary disease or psychological troubles sought help for (data not shown). Our study instrument did not include information on depression, anxiety, non-cardiac pain, gallbladder disease, cancer and gastric ulcer.

Controlling for moderate alcohol consumption and leisure-time physical activity attenuated the association in women which also became insignificant (Table VI); however, the temporal relationship between the variables is difficult to determine; we do not know whether the levels of alcohol consumption and physical activity in Sami women are due to underlying angina or have caused an excess burden of disease. It is important to note that if a sedentary lifestyle is an effect of APS, rather than a cause, the adjustment for physical activity will bias the result by reducing a true difference. The effect of leisure-time physical activity on APS in women may also reflect lower physical fitness in Sami causing an excess burden of non-cardiac pain during climbing and fast walking. In the late 1980s, Sami women had a lower level of leisure-time physical activity compared with Norwegian women. However, in terms of total physical activity, Sami men and women scored higher than did Norwegian men and women (30), which suggests that the level of leisure-time physical activity observed in this study may be due to underlying IHD in Sami women.

Less alcohol use among Sami than non-Sami has been observed in other studies (31) and a protective effect of moderate alcohol consumption has been reported, while regular ethanol consumption of more than 50 g per day tends to increase the risk for early atherogenesis (17, 32). A recent study on stable angina pectoris found a protective effect of alcohol consumption of up to 4 glasses per day (33). However, other reports suggest that the health promoting effect of moderate alcohol consumption may be confounded by other lifestyle factors associated with good health; non-drinkers compared with moderate drinkers tend to be widowed or never married, inferior with regard to SES, less likely to access health care or preventive health services, suffering from comorbid health conditions such as diabetes and hypertension and having lower levels of mental well-being (32, 34–37). Thus, the effects of moderate alcohol consumption observed in this study may be due to residual confounding and variables that we have been unable to control for.

The excess burden of angina in Sami seems to contrast with findings from the previous Finnmark studies and studies done among other Arctic indigenous peoples such as the Greenlandic Inuit, who present similar IHD morbidity rates compared to American and European populations (38). In Sweden, a few follow-up studies have been initiated showing little or no difference between Sami and non-Sami with regard to cardiovascular risk factors (39, 40), and a somewhat higher Sami mortality rate due to IHD in women (41, 42). A lower incidence rate of acute myocardial infarction has also been documented in Sami reindeer-herding women compared with Swedish women (42).

There are no obvious explanations for the ethnic difference observed in this study. The risk profiles were remarkably similar as has been presented in previous publications from the SAMINOR study (2, 43). The difference in APS is thus not explained by the conventional risk factors (Table VI). Njølstad et al. (5) found no difference in the risk of myocardial infarction (MI) between Sami and Norwegians in Finnmark, while Tverdal (10) found a reduced risk of coronary death in Sami men which was not explained by the conventional risk factors. Tynes and Haldorsen (9) reported a borderline significant slight increased risk of death due to IHD in both Sami men and women in Northern Norway, but did not assess possible intermediate contributions other than reporting a protective effect of an assumed higher intake of reindeer meat.

### Why a higher prevalence of APS in Sami than in non-Sami?

Our grasp of angina aetiology in the Sami population may be missing key factors as genetic, behavioural or environmental factors could have a substantial influence on the occurrence of disease in Sami areas. Eliassen et al. (44) found marginalization and assumed subsequent chronic stress to explain excess self-reported cardiovascular disease among Sami living in Norwegian dominated areas compared with Sami living in Sami dominated areas. Previous research has documented a consistent association between chronic stress and coronary atherosclerosis (and atherosclerotic risk factors) and these relations persist after adjusting for confounding variables and lifestyle factors (45–47). Chronic stress may be one of many explanations for some of the excess observed.

Another explanation may be an under-use of health care services in the Sami population which has been described before (48), and less satisfaction with the general practitioner (GP) service among Sami-speaking patients (49). However, in a recent study Gaski et al. (50) found no difference in health care expenditure between municipalities belonging to the administrative area of the Sami language and other municipalities. A study by Norum et al. (51) found no difference in the rate of GP referrals, when comparing the administrative area of the Sami language with a regional control group of municipalities. However, these studies may not be used to conclude any ethnic variation in referral rates and expenditure as information on individual ethnicity was not included in the analyses. Our study shows that the distribution of statin use and use of anti-hypertensive drugs do not differ among the ethnic groups; this may indicate no ethnic difference in health care utilization relevant to angina.

### Strengths

The large sample allowed a detailed analysis of angina pectoris in the Sami and non-Sami populations of rural northern Norway, and we were able to control for a number of confounding factors.

### Limitations

The main limitation in this study was the cross-sectional study design; we were unable to assess causal relationships; possible invalid temporal relationships between our outcome and explanatory variables may have hampered the ability to assess the differences in APS between Sami and non-Sami. Furthermore, the validity of our angina measures may be reduced and interpretations must hence be made with caution.

We have limited information about non-responders other than that they were younger, single and male (1); potential selection bias is thus difficult to assess. However, the differences between participants and non-participants are often important but seldom so great that studies are irrevocably undermined (52). We do not know the response rate by ethnicity; we are therefore unable to assess whether differences in participation have influenced the observed disease burden in the ethnic groups.

## Conclusions

This study revealed an excess of APS, self-reported angina, and a combination of these in Sami women and men relative to non-Sami women and men. Established risk factors explained little or none of the ethnic variation in APS. In women however, less moderate alcohol consumption and leisure-time physical activity in Sami may explain the entire ethnic difference.
